# Case Report: Identification of Polygenic Mutations by Exome Sequencing

**DOI:** 10.3389/fped.2021.689901

**Published:** 2021-10-21

**Authors:** Yanfeng Liu, Zhongshi Zheng, Qingling Zhu

**Affiliations:** ^1^Department of Endocrinology, Quanzhou Women and Children's Hospital, Quanzhou, China; ^2^Department of Children Health Care, Quanzhou Women and Children's Hospital, Quanzhou, China

**Keywords:** genetic diseases, gene mutation, distal renal tubular acidosis, *SLC4A1*, *FGFR1*, *KAT6B*

## Abstract

The discovery of rare genetic variation through different gene sequencing methods is a very challenging subject in the field of human genetics. A case of a 1-year-old boy with metabolic acidosis and hypokalemia, a small penis, growth retardation, and G-6PD deficiency was reported. Since the clinical symptoms are complex and seem uncorrelated, the authors hypothesized that the child had chromosome or gene problems, and exome sequencing (ES) was applied to samples from him and his parents. Three main locus mutations in three genes were found in the proband, including *SLC4A1, FGFR1*, and *G6PD* genes. A missense mutation (c.1766G>T, p.R589 L) was found in exon 14 of *SLC4A1* gene, which was a *de novo* mutation. Another missense mutation (c.1028 A>G, p.H343R) was found in exon 9 of *FGFR1* gene, which was also a *de novo* mutation. These findings further demonstrate the utility of ES in the diagnosis of rare diseases.

## Introduction

It is a challenging task to study the pathogenesis of genetic diseases. Exome sequencing (ES), also known as target exome capture, is a genomic analysis method that uses sequence capture technology to capture and enrich whole genome exon region DNA and conduct high-throughput sequencing. Previous studies (1) have shown that ES has high clinical practicability and is cost-effective. It has the highest diagnostic rate for children with genetic heterogeneity or characteristics that overlap multiple diseases (2). Our study reports a male boy who was found to have G-6PD deficiency and a small penis on his 15-day visit and showed growth retardation, metabolic acidosis, and hypokalemia on his 1-year second visit. The proband had no elder brother or sister; his parents were not blood relatives, and both of their phenotypes were normal. To further study the proband's disease, we sequenced the whole exome of the proband and his parents after obtaining consent from the proband's parents.

## Case Description

A male boy with normal birth weight and length was found to have G-6PD deficiency and a small penis on his 15-day first visit. Hearing screening passed in both ears. G2P1, the first fetus, was embryo stop pregnancy, with no special maternal pregnancy. At the time of his birth, his mother was 30 years old, and the father was 34 years old. There was no regular physical examination after his birth. The second visit was at 1 year of age with a slow increase in body weight, and the size and length of the penis did not change. His body length was 69.3 cm (−2.5 SD), and his weight was 6.0 kg (−4.1). Clinical evaluation showed a thin and small stature with normal appearance; penile length was 1.5 ^*^ 0.5 cm with right testicular volume test approximately 1 ml (the left was not touched), pubic hair Tanner I stage. Blood gas analysis in the outpatient department showed pH 7.226, PCO_2_ 21.60 mmHg, PO_2_ 63.50 mmHg, BE^−^ 16.70 mmol/L, BEECF^−^ 18.8 mmol/L, HCO^3−^ 8.80 mmol/L, K^+^ 2.72 mmol/L, Na^+^ 137.5 mmol/L, and Cl^−^ 109.5 mmol/L (hypokalebicarbonate acid correction, which was related to complete examinations). After repeated treatment with acidosis and oral potassium supplementation for 7 days, the blood gas basically returned to normal.

A hormonal basal study revealed low values of follicle-stimulating hormone (FSH), luteinizing hormone (LH), PREG, TESTOST, and estradiol for the proband's sex and chronological age. The gonadotropin-releasing hormone (GnRH) stimulation test elicited a prepubertal LH response with LH and FSH peaks of 0.45 and 7.10 mUI/ml, respectively. The basal value of LH was <0.07 mIU/ml. The human chorionic gonadotropin (hCG) excitation test showed a TESTOST peak of 12.36 ng/dl; the extended hCG excitation test showed that the level of TESTOST was 119.93 ng/dl, both of which suggested no obvious increase in testosterone. The results of inhibin B (INH-B) and anti-Müllerian hormone (AMH) were 21.05 pg/ml and 14.24 ng/ml, respectively, which showed poor function of testicular Sertoli cells. Drostenedione <0.44 nmol/L; dehydroepiandrosterone sulfate <0.1 μg/dl. IGF-1 was 25.0 ng/ml, and IGFBP-3 was 1.7 μg/ml, both of which were lower than in patients of the same race, sex, and age group. Plasma ammonia was elevated at 73.0 μmol/L. Citrulline was elevated at 42.71 μmol/L. A child neuropsychological development assessment suggested a low development quotient. The levels of 17-hydroxyprogesterone and hydrocortisone (COR), adrenocorticotropic hormone (ACTH), alpha-fetoprotein (AFP), carcinoembryonic antigen (CEA), and βHCG were normal. The ACTH cortisol rhythm and thyroid function were normal. The reference ranges of the test results are shown in Table 1.

**Table 1:** Laboratory test results and reference range of the child.

**Index**	**Test value**	**Reference range**
pH	7.226	7.35–7.45
PCO_2_	21.60 mmHg	34.8–44.9 mmHg
PO_2_	63.50 mmHg	79.8–100 mmHg
BE^−^	16.70 mmol/L	−3.0 to +3.0 mmol/L
BEECF^−^	18.8 mmol/L	−3.0 to +3.0 mmol/L
HCO^3−^	8.80 mmol/L	21.4–27.3 mmol/L
K^+^	2.72 mmol/L	3.5–5.3 mmol/L
Na^+^	137.5 mmol/L	136–145 mmol/L
Cl^−^	109.5 mmol/L	96–108 mmol/L
Peak of LH	0.45 mIU/ml	1.5–9.3 mIU/ml
Basal value of LH	<0.07 mIU/ml	1.5–9.3 mIU/ml
Peak of FSH	7.10 mUI/ml	1.4–18.1 mUI/ml
Peak of TESTOST	12.36 ng/dl	123.06–813.86 ng/dl
TESTOST^*^	119.93 ng/dl	94–327 pg/ml
INH-B	21.05 pg/ml	94–327 pg/ml
AMH	14.24 ng/ml	2.0–6.8 ng/ml
Drostenedione	<0.44 nmol/L	2.0–4.6 nmol/L
Dehydroepiandrosterone sulfate	<0.1 μg/dl	0.47–19.4 μg/dl
IGF-1	25.0 ng/ml	55–327 ng/ml
IGFBP-3	1.7 μg/ml	0.7–0.36 ng/ml
Plasma ammonia	73.0 μmol/L	10–47 μmol/L
Citrulline	42.71 μmol/L	5–35 μmol/L

*LH, luteinizing hormone; FSH, follicle-stimulating hormone; INH-B, inhibin B; AMH, anti-Müllerian hormone*.

* *The level of TESTOST in the extended human chorionic gonadotropin (hCG) excitation test*.

The karyotype was 46, XY. A pituitary MRI scan and enhancement showed that the pituitary gland was small and the pituitary stalk was thin, which implicated pituitary hypoplasia, and the position of the cerebellar tonsil was low. Olfactory bulb MRI showed that both olfactory tracts were asymmetric and that the olfactory sulcus was not clear. Color Doppler ultrasound of the urinary system showed that bilateral renal medulla echoes were significantly enhanced, and calcareous deposition was considered, with spermatic cord effusion on the right region and cryptorchidism on the left region. The child had normal ACTH cortisol rhythm and thyroid function.

The initial diagnoses were as follows: (1) distal renal tubular acidosis (dRTA; type I); (2) growth retardation; (3) G-6PD enzyme deficiency; and (4) congenital small penis.

After the informed consent of family members and the consent of the hospital ethics committee was obtained, the detection of trio-ES in the child and his parents was performed to detect potential variants. Sanger sequencing technology was used to verify the suspected pathogenic mutations.

## Methods for Trio-Exome Sequencing

DNA was extracted from peripheral blood, and ES was carried out on the proband and his parents. The coding exons were captured using the xGen® Exome Research Panel v1.0 (IDT). The captured fragments were sequenced using a NovaSeq 6000 sequencer (Illumina, San Diego, CA, USA) to an average depth of 100 reads per target base. The clean data were aligned to the National Center for Biotechnology Information (NCBI) human reference genome (HG19) using the Burrows–Wheeler Aligner (BWA), and variants were determined using GATK. Samtools and Pindel were used to determine single-nucleotide polymorphisms (SNPs) and indels, respectively. The clean data were filtered for further analysis according to the quality of the sequencing. For variant annotation and prediction, non-synonymous substitutions and SNPs with minor allele frequencies (MAFs) lower than 5% were filtered using SIFT. The function of mutated genes and their pathogenicity were analyzed referencing the dbSNP, 1,000 Genomes Project, ExAC, ESP, OMIM, Swiss-var, HGMD, ClinVar, and other disease databases. The variants with unknown pathogenicity of single bases were screened using Provean, SIFT, Polyphen2-HVAR, Polyphen2-HDIV, Mutationtster, and other protein structure prediction software. MaxEntScan was used to screen potential splice sites.

## Treatment

After admission, the child was treated with sodium bicarbonate 20 ml qd by intravenous injection for 7 days and 10% KCl 15.5 ml qd for 4 days, later changed to potassium citrate 2 g tid p.o. The medicine after discharge was potassium citrate 2 g tid p.o. The child has regular follow-up visits and taking medications on time without adverse drug events occurring, symptoms are well-controlled, and body length and weight are increasing steadily. The timeline for the treatment and follow-up is shown in Table 2. During the recent follow-up, parents said that the child was more active and his appetite improved significantly.

**Table 2:** Timeline for the treatment and follow-up.

**Time**	**Length (cm)**	**Weight (kg)**	**pH (7.35–7.45)**	**PCO_**2**_ (mmHg) (34.8–44.9)**	**PO_**2**_ (mmHg) (79.80–100.00)**	**BE (mmol/L) (−3.0 to 3.0)**	**HCO^**3−**^ (mmol/L) (21.4–27.3)**	**K^**+**^ (mmol/L) (3.5–5.3)**	**Cl^**−**^ (mmol/L) (96–108)**	**Treatment**
Outpatient visits 2019.6.14 (15 days)	49.0	3.3	Visited due to failing to pass newborn disease screening; G-6PD deficiency and small penis were found							Advised that the child avoid exogenous drugs such as primaquine or fava beans
Hospitalized 2020.6.19 (1 year)	63.9	6.0	7.268	20.9	53.9	−15.3	9.3	2.75	110.2	Sodium bicarbonate 20 ml qd 7 days + 10% KCl 15.5 ml qd 4 days, later changed to potassium citrate 2 g tid p.o. Medicine after discharge: potassium citrate 2 g p.o. tid
Follow-up 2020.8 (1 year 2 months)	64.5	6.5	7.41	27.6	74.7	−5.3	17.5	3.9	105.1	Potassium citrate 2 g p.o. tid
Follow-up 2020.12 (1 year 6 months)	73.5	9.0	Due to the steady increase in the length and weight of the child, and there was no complaint of discomfort; the blood gas and electrolytes were not reviewed							Potassium citrate 2 g p.o. tid
Follow-up 2021.4.20 (1 year 10 months)	78.5	11.0	7.40	30.0	76.1	−5.0	18.7	3.8	103.2	Potassium citrate 2 g p.o. tid

## Results

Seven locus mutations in seven genes were found in the proband: *SLC4A1, FGFR1, G6PD, GLI3, BCOR, FAT4*, and *KAT6B*.

A missense mutation (c.1766G>T, p.R589 L) was found in exon 14 of *SLC4A1* gene of the proband, at base 1,766, in which G was mutated to T, resulting in the mutation of the amino acid residue at position 589 from arginine to leucine. The mutation was not found in either of his parents and was a *de novo* mutation. After searching the SNP website (https://www.ncbi.nlm.nih.gov/snp/, retrieval date July 2020), we found that the R589 L mutation in the proband had not been reported before, and we named it *SLC4A1* Quanzhou according to nomenclature by city (3, 4). In addition, a missense mutation (c.1028 A>G, p.H343R) was found in exon 9 of *FGFR1* gene in the proband, which was also a *de novo* mutation. A missense mutation (c.296G>A, p.R2329H) was found in exon 3 of *KAT6B* gene, which was observed in the father, so it was a hereditary mutation. Similarly, these two mutations have not been reported. We named them *FGFR1* Quanzhou and *KAT6B* Quanzhou. The other four gene mutation sites were noted in previous reports, as shown in Table 3. The inclusion status and frequency of the three “pathogenic” genes in the database are shown in Table 4.

**Table 3:** Results of exome sequencing.

	**Chromosomal location**	**Nucleic acid change (exon no.)**	**Amino acid changes**	**RS no**.	**ACMG pathogenicity grade**	**Proband (male)**	**Father (normal)**	**Mother (normal)**	**Related diseases (OMIM), genetic pattern**
*SLC4A1*	Chr17:42333075	c.1766 (exon 14) G>T	p.R589L (NM_000342)	No items found	Pathogenic	Heterozygosity (40/75)	Wild type (0/40)	Wild type (0/630)	Renal tubular acidosis, distal (OMIM: 179800), AD
*FGFR1*	Chr8:38279344	c.1028 (exon 9) A>G	p.H343R (NM_001174064)	No items found	Likely pathogenic	Heterozygosity (32/65)	Wild type (0/41)	Wild type (0/54)	Hypogonadotropic hypogonadism 2 with or without anosmia (OMIM: 147950), AD Hartsfield syndrome (OMIM: 615465), AD
*G6PD*	ChrX:153760484	c.1466 (exon 12) G>T	p.R489L (NM_000402)	rs 72554665	Likely pathogenic	Hemizygote (49/49)	Wild type (0/52)	Heterozygosity (53/101)	Hemolytic anemia, *G6PD* deficient (OMIM: 300908), XLD
*GLI3*	Chr7:42116438	c.386 (exon 4) C>G	p.P129R (NM_000168)	rs 1276292491	Uncertain	Heterozygosity	Wild type	Heterozygosity	Pallister–Hall syndrome (OMIM: 146510), AD
*BCOR*	ChrX:39934000	c.599 (exon 4) C>T	p.T200M (NM_001123385)	rs 777945715	Uncertain	Hemizygote	Wild type	Heterozygosity	Microphthalmia, syndromic 2 (OMIM: 300166), XLD
*FAT4*	Chr4:126337745	c.6986 (exon 6) G>A	p.R2329H (NM_024582)	rs 754622270	Likely benign	Heterozygosity	Wild type	Heterozygosity	Hennekam lymphangiectasia-lymphedema syndrome 2 (OMIM: 616006), AR Van Maldergem syndrome 2 (OMIM: 615546), AR
*KAT6B*	Chr10:76602911	c.296 (exon 3) G>A	p.C99Y (NM_012330)	No items found	Uncertain	Heterozygosity	Heterozygosity	Wild type	Genitopatellar syndrome (OMIM: 606170), AD

*ACMG, American College of Medical Genetics and Genomics; AD, autosomal dominant; XLD, X-linked dominant; AR, autosomal recessive*.

**Table 4:** The inclusion status and frequency of the three “pathogenic” genes in the database.

**Gene**	**dbSNP**	**1,000 Genomes Project**	**Thousands of people in the South**	**Thousands of people in the North**	**Genome AD**	**Genome AD in East Asia**
*SLC4 A1*	Not included	Not included	Not included	Not included	Not included	Not included
*FGFR1*	Not included	Not included	Not included	Not included	Not included	Not included
*G6PD*	0.00045	0.0031	0.00	0.0063	0.0083	0.0083

**Figure 1:**
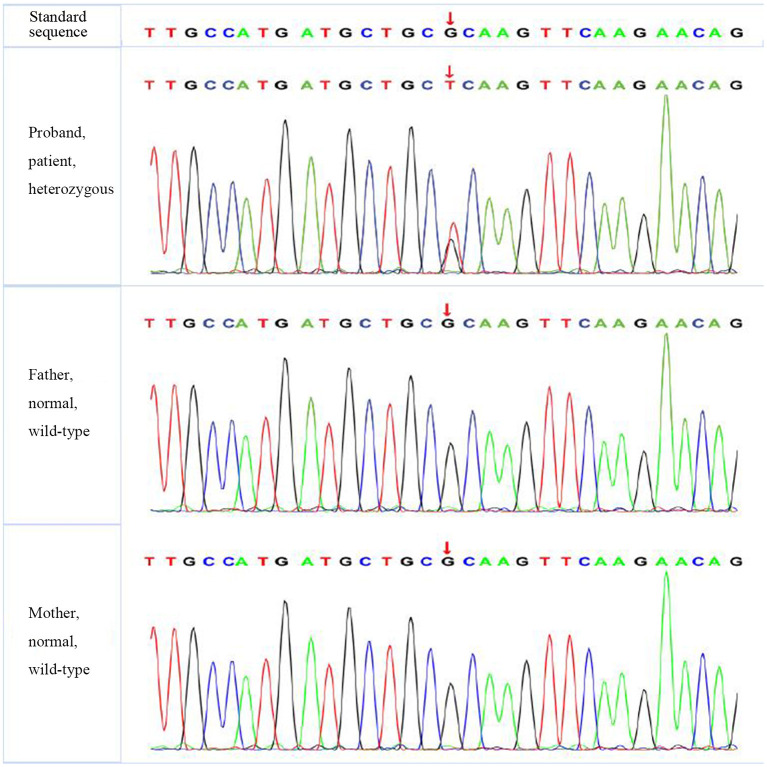
The verification results of *SLC4A1*. *SLC4A1*:c.1766 (exon 14) G>T.

**Figure 2:**
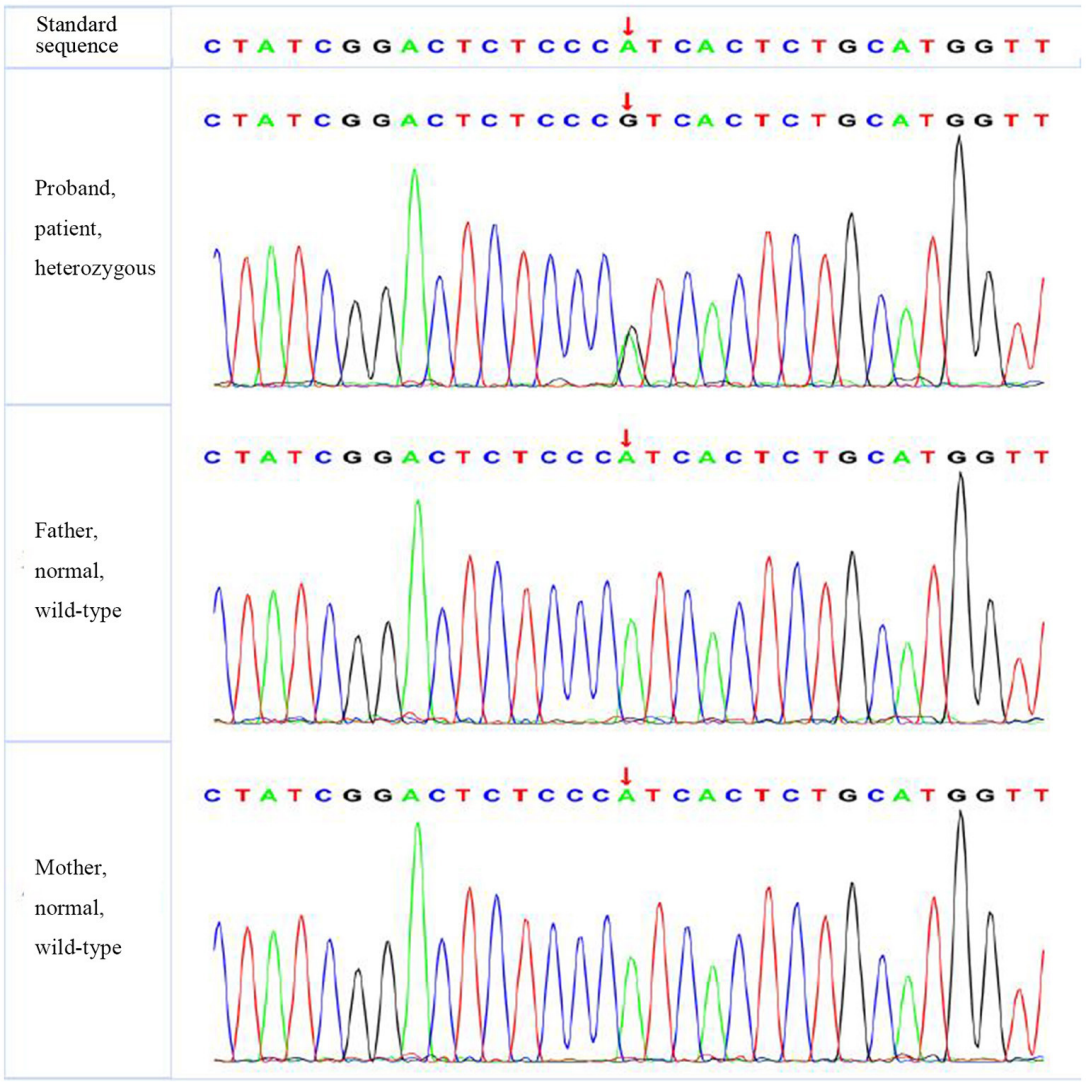
The verification results of *FGFR1*. *FGFR1*:c.1028 (exon 9) A>G.

**Figure 3:**
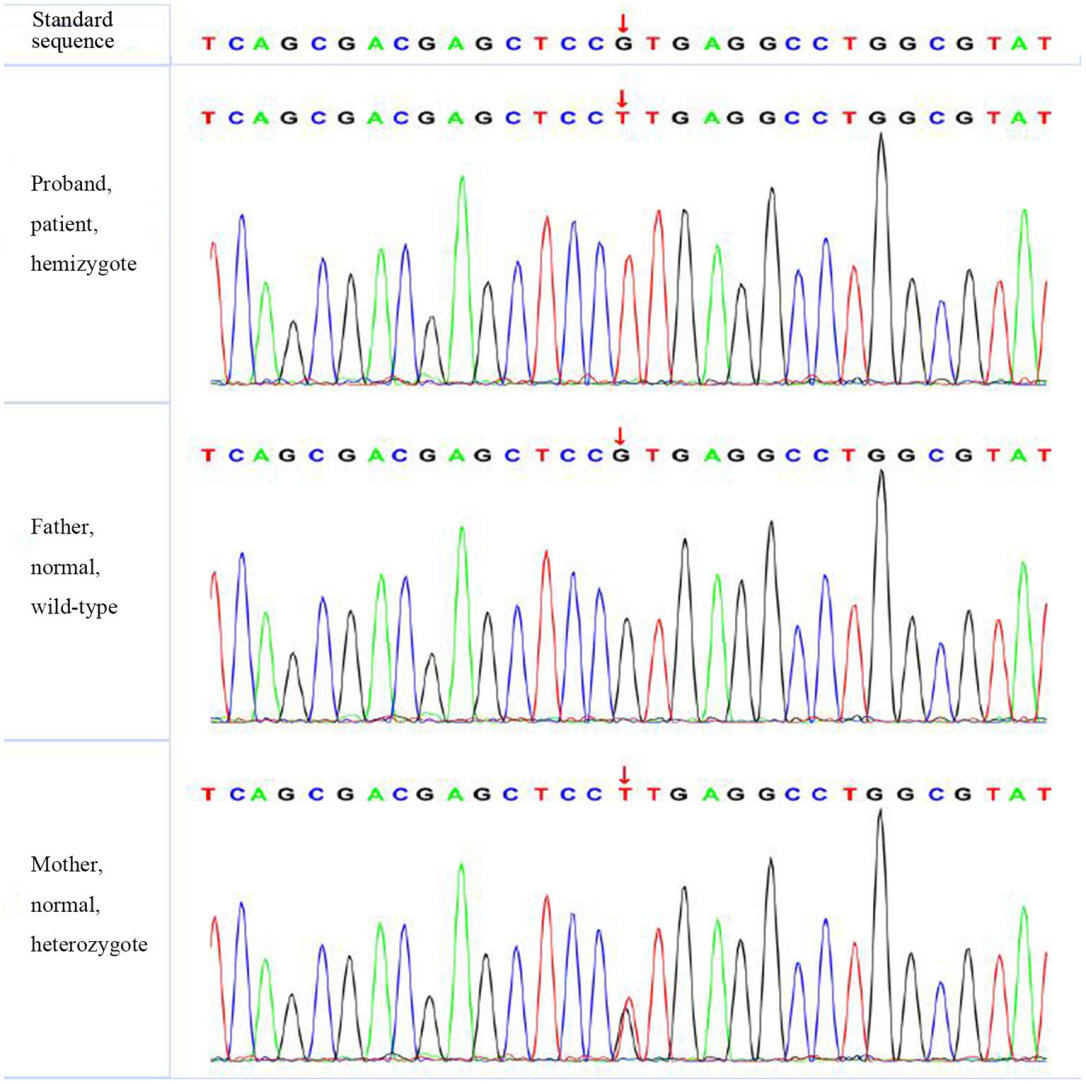
The verification results of *G6PD*. *G6PD*:c.1466 (exon 12) G>T.

According to the American College of Medical Genetics and Genomics (ACMG) guidelines (2015), *SLC4A1* gene mutation was mutated as “pathogenic (PS2 + PM1 + PM2 + PM5 + PP3),” *FGFR1* gene mutation was mutated as “likely pathogenic (PS2 + PM1 + PM2 + PP3),” and *G6PD* gene mutation was mutated as “likely pathogenic (PS1 + PM1 + PP3).” The evidence of the remaining four genes becoming pathogenic was insufficient, but possible pathogenic variation was not ruled out (Table 3). The verification results of gene mutations with high pathogenicity are shown in Figures 1–3.

## Discussion

### *SLC4A1* Gene Mutation: Pathogenic

The 17-kb *SLC4A1* gene is located on chromosome 17q21 and has 20 exons. It encodes ion exchange protein anion exchanger 1 (AE1; also called band 3 protein), first reported in 1972 (5). More than 10 mutations have been reported (6) that comprise multiple mutations of 589 loci, including R589 L, R589H, R589C, and R589S, and are considered hot spot mutations. In previous reports, *SLC4A1* gene mutation is observed with hereditary dRTA, hereditary spherocytosis, and Southeast Asian ovalocytosis.

*SLC4A1*, which encodes AE1, has two subtypes. The eAE1 subtype is expressed in red blood cells and plays a role in maintaining the normal morphology of red blood cells. The KAE1 subtype is expressed in the kidney and plays a role in transporting Cl^−^/HCO${}_{3}^{-}$. After gene mutation, the activity of Cl^−^/HCO${}_{3}^{-}$ transport was affected, and the acidification function of distal renal tubules was impaired, which led to acidosis and increased urinary pH-value. Distal renal tubular acidosis caused by *SLC4A1* gene mutation is usually caused by autosomal dominant (AD) inheritance. However, in equatorial countries such as Thailand, Malaysia, the Philippines, and Papua New Guinea, autosomal recessive inheritance is observed (7). In addition, it was reported that patients with the A858D homozygous mutation of *SLC4A1* had manifestations of both hereditary spherocytosis and dRTA (8).

The main clinical feature of dRTA is that the disorder of H^+^ secretion in the distal renal tubules causes renal acidification dysfunction, leading to high chlorine metabolic acidosis with normal anions, which is often accompanied by hypokalemia, hypercalciuria, nephrocalcinosis, and kidney stones. With the occurrence of acidosis, growth retardation and rickets can occur and have been observed in children with variants in *SLC4A1* p.R589C, p.R589H, and p.R589S. The proband presented with typical manifestations of acidosis, high blood chlorine, low blood potassium, renal calcinosis, and slow growth; and after repeated correction of acidosis and potassium supplementation, the blood gas returned to normal. Previous studies have reported that children with dRTA have a good prognosis after reasonable treatment. However, due to the possibility of deterioration of renal function in adulthood, especially after puberty, it is necessary to be vigilant and follow up regularly. In addition to *SLC4A1* gene described in this case, the hereditary pathogenic genes that have been identified to cause dRTA can also be found in *ATP6V1B1* and *ATP6V0A4* genes. Studies have found that patients with dRTA caused by *SLC4A1* gene mutations are mostly diagnosed in adolescence, while those caused by *ATP6V1B1* and *ATP6V0A4* gene mutations are more commonly identified in infancy and early childhood (9). The age of onset in this study (1 year) was significantly younger than that of *SLC4A1* gene mutations reported in previous reports.

### *FGFR1* Gene Mutation: Likely Pathogenic

Fibroblast growth factor receptor 1 (*FGFR1*), also known as acidic fibroblast growth factor receptor (aFGFR), is located on chromosome 8p12 and has 19 exons and a molecular weight of 78 kDa. Its mutation or enhanced expression can lead to changes in protein tyrosine kinase activity, thereby enhancing tyrosine kinase phosphorylation and its downstream effects. *FGFR1* is involved in the growth, differentiation, migration, apoptosis, angiogenesis, and drug resistance of tumor cells (10). Previous reports focused on the role of *FGFR1* in the field of cancer. *FGFR1* gene amplification seems to be one of the most common genetic changes in tumors and can be seen in gastric cancer, oral squamous cell carcinoma, ovarian cancer, bladder cancer, and breast cancer. *FGFR1* also plays an important role in the development of the reproductive and nervous systems. The role of nerve cell development in the embryonic stage may be related to the development of GnRH neurons and olfactory neurons. *FGFR1* protein defects can cause GnRH neuromigration and abnormal olfactory bulb development.

*FGFR1* gene mutation was reported to be associated with Kallmann syndrome (KS), which is a disease that results in idiopathic hypogonadotropic hypogonadism (IHH) combined with anosmia or hyposmia (11). Some patients with hypogonadotropic hypogonadism 2 with or without anosmia (IHH) can present specific non-reproductive phenotypes, including cryptorchidism, hypogonadotropic hypogonadism, congenital penile, olfactory loss, osteopenia, developmental delay, agenesis of the corpus callosum, bimanual synkinesis, short stature, hearing impairment, unilateral (occasionally bilateral) renal hypoplasia, cleft lip and palate, loss of teeth, and obesity (12). At present, the common causes of IHH are *FGFR1* and KAL1 gene mutations; in addition, the *FGFR1* gene is associated with Hartsfield syndrome (13).

In this case, the proband had a congenital small penis (micropenis), which means that the stretched penile length (SPL) was lower than the average of the population of the same age or the same developmental status by 2.5 standard deviations or more, accompanied by deformities such as cryptorchidism, small-volume testes, and scrotal hypoplasia. The normal value of the penis length of newborns measured by Feldman in the United States is 28–42 mm (14). Some researchers in the West have diagnosed a small penis with a penis length of <2 cm in full-term newborns. The normal penile length of full-term newborns in Asia is 26–46 mm, and it is considered that penile length <26 mm is a small penis (15).

Most *FGFR1* gene mutations were observed in male patients and were usually found in adolescence, rarely in infancy. The proband had an *FGFR1* gene heterozygous *de novo* mutation, which is consistent with the pathogenesis of AD inheritance, with clinical manifestations of congenital penile, left cryptorchidism, and growth retardation and no significant increase in testosterone in the hCG provocation test, suggesting that the genotype is consistent with the phenotype. According to the ACMG guidelines (2015), the mutation is likely to be pathogenic. In addition, *FGFR1* gene is inherited in an AD manner, so heterozygous mutations can cause disease. In this case, *FGFR1* gene of the proband is a heterozygous mutation. His parents should be wary that there is a possibility of subsequent development of IHH or Hartsfield syndrome.

### *G6PD* Gene Mutation: Likely Pathogenic

The human *G6PD* gene, located on chromosome Xq28, has 13 exons and spans 18 kb. *G6PD* gene plays a critical role in the production of ribose 5-phosphate and the generation of NADpH in the hexose monophosphate pathway. To date, there have been more than 140 mutations in *G6PD* gene found worldwide (16), and its genotype has the characteristics of regional mutations (17). Previous studies have confirmed that *G6PD* deficiency has a protective effect for malaria (18). With some environmental factors, such as infection and certain drugs and foods, *G6PD* deficiency may lead to hemolysis. It is the most common group of human enzyme deficiency syndrome and follows an X-linked incomplete dominant inheritance model.

Most *G6PD* deficiency patients are asymptomatic for life, but it can also be life-threatening, especially in children (19). The most common clinical manifestations of *G6PD* deficiency are neonatal jaundice and acute hemolytic anemia caused by exogenous drugs such as primaquine or fava beans. No persistent jaundice or clinical manifestations such as obvious hemolytic anemia were found in the neonatal period of the proband. He was discovered due to “Chinese abnormal screening of neonatal diseases” upon his first visit and was confirmed by ES by chance. The mutation was not found in the proband's father, and a heterozygous mutation was found in his mother, so he was considered to be a hemizygote.

### No Sufficient Evidence of Pathogenicity, but Variation With Pathogenicity Cannot Be Excluded

*GLI3* gene mutation was reported to be associated with Pallister–Hall syndrome (pHS; MIM 146510) (20), which was characterized by polydactyly, micropenis with undescended or hydroxyplastic testes in males, and urinary dysplasia. *BCOR* gene mutation is related to oculofaciocardiodental (OFCD) syndrome (OMIM 300166) (21), with clinical manifestations of microphthalmia, congenital cataracts, radiculomegaly, and cardiac and digital abnormalities, as well as mild developmental delay, delayed motor development, and short stature. *FAT4* gene mutation can lead to Hennekam lymphangiectasia-lymphedema syndrome 2 (OMIM 616006), and Van Maldergem syndrome 2 (OMIM 615546) (22). The former is mainly manifested as lymphedema, claudication dementia, and developmental delay, while the latter is characterized by intellectual disability, and some may suffer from renal hyperplasia. Both of these diseases may have intellectual disability and developmental delay. In addition, small kidney and genital abnormalities, including micropenis, cryptorchidism, and double scrotum, have been observed in Van Maldergem syndrome (23). *KAT6B* gene mutation is relevant to genitopatellar syndrome (OMIM 606170) (24), with main manifestations of patellar loss, congenital flexion contracture of lower limbs, psychomotor disorders, and abnormalities of external genitalia and kidneys.

In our study, the proband had the clinical manifestations of delayed growth and development, slow height and weight gain, cryptorchidism, and micropenis, which may have a certain degree of crossover with the four gene-related diseases mentioned above. According to the ACMG guidelines, the four gene mutations were identified as having insufficient evidence of pathogenicity, but possible pathogenic variation was not excluded.

### Polygenic Mutation

Many human diseases are likely caused by single-gene and/or multiple-gene mutations, which regulate the occurrence and development of diseases through gene interactions, thereby affecting human health (25). It is not uncommon to have polygenic mutations in the same patient. However, the mechanism of these diseases and the principle of gene interaction are not clear. Multiple mutations tend to have different effects on genes compared with single mutations.

Multiple-gene mutations associated with the same disease have been reported, with incidences of digenic and trigenic variants of 8/48 (16.7%) and 1/48 (2.1%), respectively (26). Various gene mutations may work together in the same pathway to affect the phenotype of the disease, while they may have no or only minimal effect on the phenotype when these heterozygous mutations exist alone.

Multiple mutations in the same gene, also known as compound mutations, have been found to be associated with various genetic diseases. Some studies have also reported that the combined effect of multiple mutations in the same gene leads to more serious cardiovascular disease than a single mutation (27). They believe that these multiple-gene mutations have additive effects on cardiovascular pathology and function and highlight the importance of basic molecular, cellular, and animal model studies in elucidating key pathogenic pathways. However, some studies suggest that multigene mutations can either aggravate the development of the disease or lighten it; the latter is known as a compensatory mutation (28).

Our case is different from the above two cases. In this case, seven gene mutations were detected by the whole-exome test, among which one was pathogenic and two were likely to be pathogenic. The other four genes with insufficient evidence need to be further confirmed. These gene mutations are not the cause of the same disease, and there is no correlation among them that has been reported. However, the cause of this patient's polygenic mutation is unclear. The existence of multiple mutations directly affects the strategy of gene diagnosis. It is important to study the combined effect of multiple mutations. Thus, we look forward to more research on the mechanism of multigene mutations.

Inevitably, there were some limitations to this study. First, we did not have the capacity to conduct functional research; thus, missense mutations could not be verified. In addition, this article focuses on the results of exon detection of this rare polygenic mutation; thus, only the details related to the clinical phenotype and treatment of this child were discussed, not all the relevant details of the related diseases.

The discovery of rare genetic variation through different gene sequencing methods is a very challenging subject in the field of human genetics. Our research confirms the role of ES in the diagnosis of dRTA. With the help of ES, case detection, and diagnosis become easier.

## Conclusions

The child needs to take bismuth potassium citrate 2 g qd orally for life, and his parents should be wary of the possibility of subsequent development of IHH or Hartsfield syndrome. In addition, the child should avoid exogenous drugs such as primaquine or fava beans. Our case expanded the gene mutation spectrum and enriched the human gene mutation library. With the expansion of newborn screening programs, including for citrullinemia and *G6PD* deficiency, numerous asymptomatic infants have been identified, aiding in treatment decisions and genetic counseling. In addition, our study extends the mutation spectrum of dRTA and is helpful in early molecular diagnoses of dRTA. In clinical work, attention should be given to the follow-up of multiple-gene mutations, especially asymptomatic infants identified in infancy.

## Data Availability Statement

The original contributions presented in the study are included in the article/supplementary material, further inquiries can be directed to the corresponding author/s.

## Ethics Statement

The studies involving human participants were reviewed and approved by Quanzhou Women and Children's Hospital Ethics Committee. Written informed consent to participate in this study was provided by the participants' legal guardian/next of kin. Written informed consent was obtained from the participants' legal guardian/next of kin for the publication of this case report.

## Author Contributions

YL and ZZ were involved in two sessions with the patient. QZ analyzed the sequencing data and performed the literature research. QZ wrote the first draft of the report, which was edited by all authors. All authors reviewed and agreed to the final version of the article. QZ had the final responsibility for the decision to submit for publication.

## Conflict of Interest

The authors declare that the research was conducted in the absence of any commercial or financial relationships that could be construed as a potential conflict of interest.

## Publisher's Note

All claims expressed in this article are solely those of the authors and do not necessarily represent those of their affiliated organizations, or those of the publisher, the editors and the reviewers. Any product that may be evaluated in this article, or claim that may be made by its manufacturer, is not guaranteed or endorsed by the publisher.

## References

[1] Tan TY Dillon OJ Stark Z Schofield D Alam K Shrestha R. Diagnostic impact and cost-effectiveness of whole-exome sequencing for ambulant children with suspected monogenic conditions. JAMA Pediatr. (2017) 171:855–62. 10.1001/jamapediatrics.2017.1755 28759686 PMC5710405

[2] Theunissen TE Sallevelt SC Hellebrekers DM De Koning B Hendrickx AT van den Bosch BJ. Rapid resolution of blended or composite multigenic disease in infants by whole-exome sequencing. J. Pediatrics. (2017) 182:371.e2–4.e2. 10.1016/j.jpeds.2016.12.032 28081892

[3] Jomoui W Panichchob P Rujirachaivej P Panyasai S Tepakhan W. Coinheritance of Hb A 2-Melbourne (HBD: c.130G>A) and Hb E (HBB: c.79G>A) in Laos and simultaneous high resolution melt detection of Hb A 2-Melbourne and Hb A 2-Lampang (HBD: c.142G>A) in a single tube. Hemoglobin. (2019) 43: 214–7. 10.1080/03630269.2019.1651332 31450984

[4] Shao L Yan X Dong Q Lang Y Yue S Miao Z. novel *SLC4A1* variant in an autosomal dominant distal renal tubular acidosis family with a severe phenotype. Endocrine. (2010) 37:473–8. 10.1007/s12020-010-9340-6 20960171

[5] Richards P Wrong OM. Dominant inheritance in a family with familial renal tubular acidosis. Lancet. (1972) 2:998–9. 10.1016/s0140-6736(72)92406-3 4116984

[6] Deejai N Wisanuyotin S Nettuwakul C Khositseth S Sawasdee N Saetai K. Molecular diagnosis of solute carrier family 4 member 1 (*SLC4A1*) mutation-related autosomal recessive distal renal tubular acidosis. Lab Med. (2019) 50:78–86. 10.1093/labmed/lmy051 30124986

[7] Khositseth S Bruce LJ Walsh SB Bawazir WM Ogle GD Unwin RJ. Tropical distal renal tubular acidosis: clinical and epidemiological studies in 78 patients. QJM. (2012) 105:861–77. 10.1093/qjmed/hcs139 22919024

[8] Shmukler BE Kedar PS Warang P Desai M Madkaikar M Ghosh K. Hemolytic anemia and distal renal tubular acidosis in two Indian patients homozygous for *SLC4A1*/AE1 mutation A858D. Am J Hematol. (2010) 85:824–8. 10.1002/ajh.21836 20799361 PMC3517738

[9] Palazzo V Provenzano A Becherucci F Sansavini G Mazzinghi B Orlandini V. The genetic and clinical spectrum of a large cohort of patients with distal renal tubular acidosis. Kidney Int. (2017) 91:1243–55. 10.1016/j.kint.2016.12.017 28233610

[10] Yu T Yang Y Liu Y Zhang Y Xu H Li M. A *FGFR1* inhibitor patent review: progress since 2010. Expert Opin Ther Pat. (2017) 27:439–54. 10.1080/13543776.2017.1272574 27976968

[11] Choi JH Oh A Lee Y Kim GH Yoo HW. Functional characteristics of novel *FGFR1* mutations in patients with isolated gonadotropin-releasing hormone deficiency. Exp Clin Endocrinol Diabetes. (2020) 129:457–63. 10.1055/a-1151-4800 32485746

[12] Hardelin JP Dodé C. The complex genetics of Kallmann syndrome: KAL1, *FGFR1*, FGF8, PROKR2, PROK2, et al. Sex Dev. (2008) 2:181–93. 10.1159/000152034 18987492

[13] Courage C Jackson CB Owczarek-Lipska M Jamsheer A Sowińska-Seidler A Piotrowicz M. Novel synonymous and missense variants in *FGFR1* causing Hartsfield syndrome. Am J Med Genet A. (2019) 179:2447–53. 10.1002/ajmg.a.61354 31512363

[14] Feldman KW Smith DW. Fetal phallic growth and penile standards for newborn male infants. J Pediatr. (1975) 86:395–8. 10.1016/s0022-3476(75)80969-3 1113226

[15] Lian WB Lee WR Ho LY. Penile length of newborns in Singapore. J Pediatr Endocrinol Metab. (2000) 13:55–62. 10.1515/jpem.2000.13.1.55 10689638

[16] Cappellini MD Fiorelli G. Glucose-6-phosphate dehydrogenase deficiency. Lancet. (2008) 371:64–74. 10.1016/s0140-6736(08)60073-2 18177777

[17] Lo E Zhong D Raya B Pestana K Koepfli C Lee MC. Prevalence and distribution of *G6PD* deficiency: implication for the use of primaquine in malaria treatment in Ethiopia. Malar J. (2019) 18:340. 10.1186/s12936-019-2981-x 31590661 PMC6781416

[18] Min-Oo G Gros P. Erythrocyte variants and the nature of their malaria protective effect. Cell Microbiol. (2005) 7:753–63. 10.1111/j.1462-5822.2005.00524.x 15888079

[19] Luzzatto L Arese P. Favism and glucose-6-phosphate dehydrogenase deficiency. N Engl J Med. (2018) 378:60–71. 10.1056/nejmra1708111 29298156

[20] Kariminejad A Ghaderi-Sohi S Keshavarz E Hashemi SA Parsimehr E Szenker-Ravi E. A *GLI3* variant leading to polydactyly in heterozygotes and Pallister-Hall-like syndrome in a homozygote. Clin Genet. (2020) 97:915–9. 10.1111/cge.13730 32112393

[21] Ng D Thakker N Corcoran CM Donnai D Perveen R Schneider A. Oculofaciocardiodental and Lenz microphthalmia syndromes result from distinct classes of mutations in *BCOR*. Nat Genet. (2004) 36:411–6. 10.1038/ng1321 15004558

[22] Alders M Al-Gazali L Cordeiro I Dallapiccola B Garavelli L Tuysuz B. Hennekam syndrome can be caused by FAT4 mutations and be allelic to van Maldergem syndrome. Hum Genet. (2014) 133:1161–7. 10.1007/s00439-014-1456-y 24913602

[23] Neuhann TM Müller D Hackmann K Holzinger S Schrock E Di Donato N. further patient with van Maldergem syndrome. Eur J Med Genet. (2012) 55:423–8. 10.1016/j.ejmg.2012.02.012 22469822

[24] Okano S Miyamoto A Fukuda I Tanaka H Hata K Kaname T. Genitopatellar syndrome: the first reported case in Japan. Hum Genome Var. (2018) 5:8. 10.1038/s41439-018-0010-1 29899993 PMC5972145

[25] Loeb KR Loeb LA. Significance of multiple mutations in cancer. Carcinogenesis. (2000) 21:379–85. 10.1093/carcin/21.3.379 10688858

[26] Quaynor SD Bosley ME2 Duckworth CG Porter KR Kim SH Kim HG. Targeted next generation sequencing approach identifies eighteen new candidate genes in normosmic hypogonadotropic hypogonadism and Kallmann syndrome. Mol Cell Endocrinol. (2016) 437:86–96. 10.1016/j.mce.2016.08.007 27502037

[27] Kelly M Semsarian C. Multiple mutations in genetic cardiovascular disease: a marker of disease severity? Circ Cardiovasc Genet. (2009) 2:182–90. 10.1161/CIRCGENETICS.108.836478 20031583

[28] Liu M Watson LT Zhang L. Predicting the combined effect of multiple genetic variants. Hum Genomics. (2015) 9:18. 10.1186/s40246-015-0040-4 26223264 PMC4520001

